# Inequities in breast cancer outcomes in Chile: An analysis of case fatality ratios and survival rates (2007–2018)

**DOI:** 10.1371/journal.pone.0325252

**Published:** 2025-09-29

**Authors:** Benjamín Madariaga, Susana Mondschein, Soledad Torres

**Affiliations:** 1 Department of Mathematical Engineering, University of Chile, Santiago, Chile; 2 Department of Industrial Engineering, University of Chile, Santiago, Chile; 3 Instituto Sistemas Complejos de Ingeniería (ISCI), Santiago, Chile; 4 Center for Cancer Prevention and Control (CECAN), Santiago, Chile; 5 Clínica Meds, Santiago, Chile; Universidad Católica Sedes Sapientiae: Universidad Catolica Sedes Sapientiae, PERU

## Abstract

Breast cancer is a leading cause of illness and death among women in Chile, yet national data on health outcomes remain limited in the absence of a cancer registry. This observational study examines disparities in breast cancer case fatality ratios and survival rates by health insurance provider and geographic region using national hospital discharge and mortality databases from 2007 to 2018. We analyzed 58,254 hospital discharges and 16,615 deaths related to breast cancer. Case fatality and survival estimates were computed using crude ratios, Kaplan-Meier methods, and Cox proportional hazards models. Nationally, the average case fatality ratio was 26.8 percent. Patients in the public health insurance system had significantly higher fatality ratio (27.5 percent) than those in the private system (15.7 percent). One- and five-year survival rates were lower for publicly insured patients (93.4 percent and 80.8 percent) than for privately insured patients (97.3 percent and 90.2 percent). Within the public system, survival varied by income-based segment, with the lowest rates among the most socioeconomically disadvantaged group. Patients in the Metropolitan Region showed better survival compared to those living in other regions. Cox regression analysis confirmed that health insurance type, age, year of diagnosis, and region of residence were significant predictors of survival. These findings suggest that, despite universal health guarantees in Chile, meaningful inequities in breast cancer outcomes persist. The methodology used in this study relies on administrative data and can be applied in other countries or regions with access to comparable hospital discharge and mortality records, supporting broader efforts to monitor and reduce healthcare disparities.

## Introduction

Breast cancer (BC) is the most common female cancer worldwide, and Chile is not an exception; new BC cases have increased significantly from 3,785 cases in 2007 to 5,435 in 2018 [1]. In 2020, age-standardized world breast cancer incidence and mortality rates were 47.8/100,000 and 13.6/100,000 women, respectively [2]; the figures reported for Chile by the Ministry of Health (Minsal) for 2018 were 44.1/100,000 and 11.3/100,000 women for incidence and mortality rates, respectively [3,4].

Chile has a mixed health insurance system composed of a public subsystem, administered by the National Health Fund (FONASA), and a private subsystem, composed of Private Health Insurance Institutions (ISAPREs). As of 2024, approximately 77% to 83% of the population is affiliated with FONASA, while 16% to 20% are enrolled in ISAPREs; the remaining 3% to 5% are covered by other systems, such as the Armed Forces or remain uninsured.

FONASA classifies its beneficiaries into four income-based groups, which determine the level of copayment and access to services: Group A includes individuals with no formal income or in vulnerable conditions and provides free access to care; Group B includes low-income earners and also provides free care; Group C corresponds to middle-income individuals who must pay a 10% copayment; and Group D includes higher-income individuals with a 20% copayment. All FONASA beneficiaries can access services through the institutional modality (public hospitals and clinics), while groups B, C, and D may also use the Free Choice Modality (Modalidad de Libre Elección, MLE) to receive care from private providers under agreement with FONASA, with varying levels of subsidy. In contrast, ISAPREs operate on a model of individual insurance plans, offering access to private healthcare facilities with variable premiums and copayments [5]. This structure reflects Chile’s approach to balancing public universal coverage with private alternatives, while also highlighting persistent disparities in access and financial protection across income groups.

Thus, the selection of a healthcare provider is primarily determined by an individual’s economic income [6,7]; as a consequence, profound inequalities in terms of quality of life, education, and other factors have existed between users of private and public affiliates. In particular, healthcare indicators have been significantly better for those with private health insurance compared to those for publicly insured patients. For example, in 2017, 27.8% of FONASA affiliates reported having problems obtaining healthcare, while this percentage decreased to 12.6% for ISAPRE affiliates [8].

Consequently, as an effort to provide better healthcare services for all citizens, in 2004, Chile implemented a comprehensive health reform, the Explicit Health Guarantees (GES), which aimed to achieve a more equitable and fairer system [9]. This reform aimed to ensure all Chilean citizens’ access, quality, opportunity, and financial protection, requiring care for the most common pathologies. Due to budget constraints, the program started in 2005 with 25 pathologies, including breast cancer (for people aged 15 years and over with suspected, diagnosed, or recurrent breast cancer), and has gradually grown to cover 85 pathologies currently. As stated by the Minister of Health, Pedro García, in 2005, the GES plan will represent a qualitative leap toward greater equity in the healthcare sector. The country will enter “a path of no return toward greater dignity for the citizens of our country, and for that, we are proud” [10]. Twenty years after its implementation, there is substantial evidence that the GES plan has partially achieved its goal of improving opportunities, with 92.8% of services requested in 2017 fulfilled within the expected time [11]. The plan is also constantly monitoring the quality of healthcare centers [12]. Regarding access, among people from FONASA who received healthcare in 2017, 27.8% reported having received healthcare with some access difficulty, while the remaining 72.2% received healthcare without access difficulties. The exact figures for ISAPRE affiliates are 12.6% and 87.4%, respectively [8].

However, to the best of our knowledge, no studies have demonstrated the effectiveness of the GES plan in terms of healthcare outcomes, including survival and case fatality ratios. In particular, the focus of this paper is to study whether the inclusion of breast cancer in the GES program, including these four guarantees for all women, has resulted in a reduction (or elimination) of the gap in health outcomes, including case fatality ratios and survival rates among Chilean women.

Thus, the primary goal of this paper is to study inequities in breast cancer outcomes, such as fatality and survival rates, in Chilean women stratified by type of healthcare insurance provider and geographical area. To achieve this objective, we used two public anonymized datasets compiled by the Department of Health Statistics and Information (DEIS) of the Ministry of Health following the methodology used in [1], along with incidence and mortality rates for the 2007–2018 period, also obtained from [1].

## Materials and methods

### Ethics statement

This work utilized publicly available data from the Chilean Ministry of Health, specifically from the Department of Health Statistics and Information. All data were protected, and personal information was anonymized; therefore, no consent from participants was needed.

### Data

DEIS provided two public anonymized databases. The first is the National Death Registry, which includes 2,549,800 deaths from January 1990 to December 2018. For each death entry in the registry, the patient’s ID (identifying code), date of birth, date of death, gender, town and region of residence, marital status, occupation, and cause of death code according to the International Statistical Classification of Diseases and Related Health Problems (CDI-10) were available. The second database includes all discharges from the country’s public and private healthcare facilities, comprising 32,443,591 registries from January 2001 to December 2020. Each registry has 39 fields, including the patient’s ID (identical to the national death database), date of birth, gender, town and region of residence, ethnicity, health insurance, length of stay, condition at discharge, and primary and secondary diagnoses according to the ICD-10 classification, among others.

These two datasets were processed as in the study [1]. This processing resulted in a total of 58,254 and 16,615 BC hospital discharges and deaths for the period 2007–2018. These registries were used for the survival analysis.

### Methods

The annual case fatality ratio (CFR) is defined as the ratio of the number of women who died from breast cancer in a given year to the number of new breast cancer cases diagnosed during that same year. Consequently, the numerator and denominator represent different cohorts of individuals. CFRs were calculated for each year and strata (region and healthcare system) as *Crude Mortality/Crude Incidence*. Incidence and mortality rates were obtained from [1].

For the survival analysis, we used the standard estimator of the survival function proposed by Kaplan and Meier [13], namely, the product-limit estimator, considering right-censored data. In our study, censored data consisted of breast cancer patients who either died of other causes during the timeline of the research or survived from then on. To estimate survival, we considered time-on-study survival as our variable of interest, or equivalently, the time from the first breast cancer diagnosis to death. Without loss of generality, we measured time in months, and therefore, the survival time was recorded as months difference; for example, a patient diagnosed on 1/1/2007 who died on 01/31/2007 had a one-month survival, while a patient diagnosed on 1/31/2007 who died on 2/01/2007 had a two-month survival. The Kaplan-Meier survival curves are shown with their 95% confidence intervals (p<0.05) in the Results section.

Finally, we used the Cox proportional hazards model to study the effects of covariates [14]. We used health insurance provider, age, year of discharge, residence in the metropolitan region, and FONASA benefit segments as covariates. These covariates were processed, transforming the categorical ones into dummy variables, resulting in 13 covariates from which to select those relevant to the model. In the above process, patients with an unknown health insurance system were assigned a separate dummy variable indicating the absence of classification into any known healthcare system. Consequently, these patients were included in the calculation of national-level survival curves but excluded from survival curves specific to the public and private healthcare systems. The selection was based on Akaike’s information criterion [15] and on the p-value for each variable’s significance (p<0.001), while making sure that all variables in the model were independent. See S1 Appendix for a description of the model selection procedure.

Because the dataset used to estimate case fatality and survival is very large, statistical tests have high power and can yield p<0.05 even for effects that are negligible in practice. To reduce the risk of rejecting the null hypothesis based on such trivial differences, we adopt a more stringent significance threshold (p<0.001).

## Results

### Case fatality ratios

**Fig 1:**
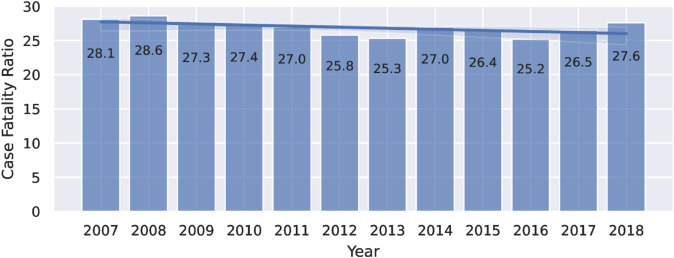
Case fatality ratio (%) by year for the 2007–2018 period. **Alt text:** Bar graph showing case fatality ratios from 2007 until 2018. All values sit between 28.6% and 25.3%. There is a linear regression plotted over the bars; the plotted regression is slightly decreasing but almost completely horizontal.

Case fatality ratios (%) by year for the 2007–2018 period are shown in Fig 1. The overall fatality ratio remained relatively constant over time, with a mean of 26.8 and a standard deviation of 1.1.

Table 1 shows case fatality ratios for women affiliated with the private and public health systems. As stated in [1], there is a set of women from the death database for whom there is no information about their healthcare provider. This problem does not occur for the incidence rate estimations because the discharge database provides information on the patient’s healthcare insurance. As fatality ratios are calculated based on crude mortality and incidence rates, this missing information also affects CFRs reported by health insurers. Assuming that the death registries for which this information was unavailable are unbiased regarding healthcare insurance, the case fatality ratios shown in Table 1 are reasonable estimates. Women affiliated with ISAPREs have a considerably lower fatality ratio during the period under study, with an average of 15.7 compared to 27.5 for women affiliated with the FONASA.

**Table 1:** Case fatality ratio (%) by health insurance.

Year	ISAPRE	FONASA
	(private)	(public)
2007	23.3	26.5
2008	19.3	26.4
2009	12.7	28.1
2010	14.3	27.4
2011	16.1	27.1
2012	13.5	26.6
2013	16.0	26.0
2014	13.6	29.2
2015	16.1	27.2
2016	12.2	27.9
2017	15.5	28.1
2018	15.3	29.0
Mean (std)	15.7 (3.1)	27.5 (1.0)

The Mann-Kendall test was applied under the null hypothesis of no trend (increasing or decreasing). The test yielded p-values of 0.1 and 0.6 for FONASA and ISAPRE respectively, supporting the null hypothesis in both cases. Therefore, neither of the systems showed a statistically significant trend(increasing or decreasing) (p<0.001). However, the CFRs from ISAPRE affiliates had more significant fluctuations, with a standard deviation of 3.1, while FONASA had a standard deviation of 1.0.

**Fig 2:**
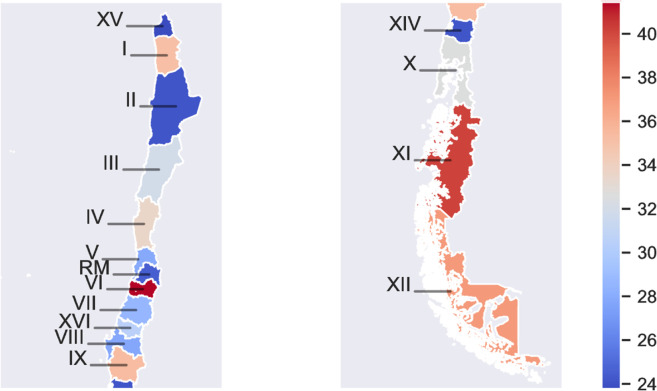
Mean case fatality ratio (%) over 2007-2018 by geographical region. Detailed fatality values are in Table 2. Shapefiles by region obtained from Chile’s National Congress Library (https://www.bcn.cl/siit/mapas_vectoriales/index_html). **Alt text:** Figure shows a map of Chile segmented by regions. Each region has a distinct color, as indicated on a color map.

Fig 2 and Table 2 show considerable differences in case fatality ratios across regions. The regions with the highest ratios are O’Higgins (VI) and Aysén (XI), with fatality ratios of 41.4% and 40.1%, respectively. On the other hand, the regions with the lowest ratios are Metropolitan (RM), Los Ríos (XIV), Antofagasta (II), and Arica and Parinacota (XV), with average fatality ratio of 24.5%, 24.2%, 24.1%, and 23.8%, respectively. The fatality ratio of the other regions are between 27.8% and 37.1%.

**Table 2:** Average case fatality ratio (%) over the period 2007–2018 by region.

Region	Region Name	Case Fatality Ratio
XV	Arica y Parinacota	23.8
I	Tarapacá	35.1
II	Antofagasta	24.1
III	Atacama	31.9
IV	Coquimbo	33.5
V	Valparaíso	27.9
RM	Metropolitana de Santiago	24.5
VI	Libertador General Bernardo O’Higgins	41.4
VII	Maule	28.6
XVI	Ñuble	30.5
VIII	Biobío	27.8
IX	La Araucanía	35.3
XIV	Los Ríos	24.2
X	Los Lagos	32.5
XI	Aysén del General Carlos Ibáñez del Campo	40.1
XII	Magallanes y de la Antártica Chilena	37.1

Table displays the mean case fatality ratio over the period 2007–2018 for each of the 16 Chilean regions. They are sorted from north to south.

### Survival rates

From the set of 58,254 patients who had a first discharge during the study period, 19 patients had inconsistencies when matching both databases; they were "discharged" after their death date. Therefore, these records were eliminated from the survival analysis, resulting in a survival database of 58,235 records.

#### Kaplan–Meier estimations

**Fig 3:**
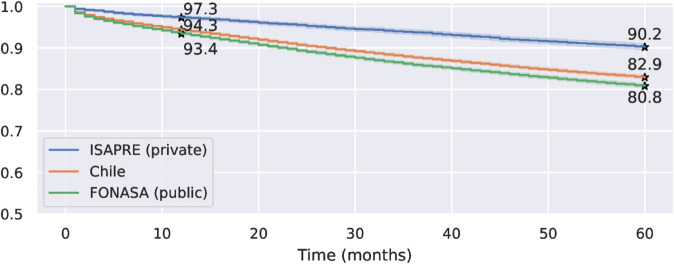
Kaplan–Meier survival curves for Chilean women separated by health insurance system (public vs. private). The number of patients at risk after 60 months is 4.895, 25.017, and 18.179 for ISAPRE, Chile, and FONASA curves, respectively. Detailed numbers on survival curves are in S1 Table, S2 Table, and S3 Table. **Alt text:** Figure shows three stepped Kaplan–Meier curves, showing the estimated survival rate over time from 0 to 60 months. These three curves are the ISAPRE (private), Chile, and FONASA (public) survival rates. The three curves are descending, starting with a survival of 100% at time 0. The ISAPRE curve is always above the other two curves, and the FONASA curve is always below. The estimated one-year and five-year survival rates are highlighted for each curve.

The Kaplan-Meier estimator was computed considering 58,235 observations, of which 50,126 were right-censored. Fig 3 shows the survival rates for all patients during the study period, separated by private and public healthcare insurance systems. The log-rank test confirmed that the survival curves for private and public insured patients, as well as all women, are significantly different (p < 0.001). The logrank test is used to assess the null hypothesis that there is no difference in survival among women from different cohorts. The test rejected the null hypothesis (p<0.001) for both privately and publicly insured patients, as well as for all women combined; therefore, the differences are statistically significant.

The estimated one-year survival rates 95% confidence intervals are [0.934 ± 0.002] for FONASA, [0.973 ± 0.003] for ISAPRE patients, and [0.943 ± 0.002] when considering all women. The 95% confidence intervals for the five-year survival rate were [0.808 ± 0.004], [0.902 ± 0.007], and [0.829 ± 0.004] for FONASA, ISAPRE, and all women, respectively. Thus, women in the private system have a five-year survival rate that is 12% higher than that for women in the public system.

**Fig 4:**
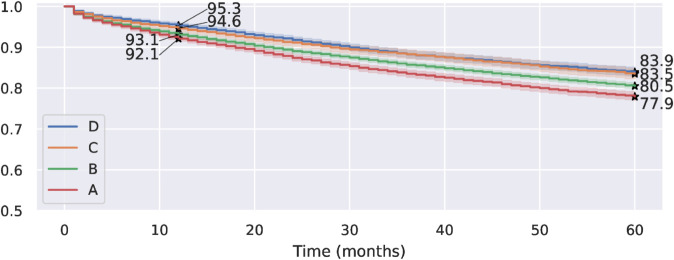
Kaplan–Meier survival curves for FONASA patients by benefit segment. The number of patients at risk after 60 months is 3.093, 2.183, 8.141, and 4.688 for segments D, C, B, and A, respectively. **Alt text:** Figure shows four stepped Kaplan–Meier curves, showing the estimated survival rate over time from 0 to 60 months. These four curves represent the survival rates for FONASA patients in segments A, B, C, and D. The curves are descending, starting with a 100% survival rate at time 0. The A-segment curve is below the other three curves at all times; the B-segment curve is below the C and D segments at all times; and the C and D curves are very close to each other, with the D curve over the C curve at times, but without a clear dominance. The estimated one-year and five-year survival rates are highlighted for each curve.

A similar result was obtained when analyzing the survival curves for patients in each segment within the public health system, as shown in Fig 4. Women with segments C and D health insurance benefits have similar survival rates, which are higher than those for women in FONASA segments A and B. Women in segment A have the worst survival rates out of the four groups. The 5-year survival rates are 0.839, 0.835, 0.805, and 0.779 for benefit segments D, C, B, and A, respectively. We notice that all these survival rates are lower than those for women in the private system.

**Fig 5:**
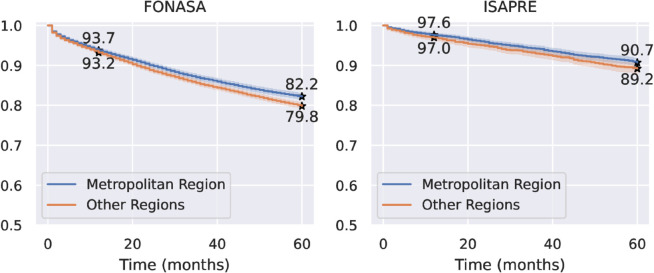
Kaplan–Meier survival curves for women in FONASA and ISAPRE, separated by their region of residence. The number of patients at risk after 60 months for FONASA is 7.676 for the Metropolitan region and 10.470 for other regions. The number of patients at risk after 60 months for ISAPRE is 2.976 for the Metropolitan region and 1.902 for other regions. **Alt text:** Figure shows two graphs. Each graph comprises two stepped Kaplan–Meier curves, showing the estimated survival rate over time from 0 to 60 months. The left graph shows survival curves for women in FONASA, and the right graph shows survival curves for women in ISAPRE. The two curves in each graph are the survival rates for patients from the metropolitan area and other regions. The two curves from both graphs are descending, starting with a survival of 100% at time 0. The Metropolitan region curve is always above the curves of the other regions in both graphs. Both curves associated with ISAPRE show higher survival rates than the curves associated with FONASA. The estimated one-year and five-year survival rates are highlighted for each curve.

Women from the Metropolitan region have a higher survival rate than women from other regions, both for women in FONASA and ISAPRE. We observe this in Fig 5, with five-year survival rates of [0.822 ± 0.006] and [0.798 ± 0.006] for women in FONASA from the Metropolitan region and other regions, respectively.The five-year survival rates for women in ISAPRE for the Metropolitan region and other regions are [0.907 ± 0.008] and [0.892 ± 0.012], respectively. The two curves in each graph are significantly different (p<0.05) The log-rank test was used to evaluate differences in survival between healthcare systems. We found that the survival of women in FONASA from the Metropolitan region versus other regions is significantly different (p<0.001). In contrast, the survival difference for women in ISAPRE between the Metropolitan region and other regions does not reach the established significance threshold, but may still be clinically relevant (p = 0.01).

#### The Cox proportional hazards model.

Using the procedure described in S1 Appendix, we estimated a Cox proportional hazards model with 8 selected variables out of 13 considered. The results are summarized in Table 3, where the second column contains the coefficient, the third its associated 95% confidence interval, and the fourth column shows the p-value for the null hypothesis corresponding to the equality of the base and the affected covariate. The dummy variable associated with the Armed Forces and unknown healthcare system were not selected due to their p-value (>0.001), meaning that these variables are not statistically significant to the model.

**Table 3:** Results for the Cox proportional hazard regression model.

Variable	Hazard Ratio	Confidence interval	p value
Year of diagnosis	-0.051	[-0.058,-0.043]	<0.001
Age	-6.484	[-7.387,-5.58]	<0.001
Squared age	7.136	[6.39,7.882]	<0.001
ISAPRE	-0.286	[-0.397,-0.175]	<0.001
FONASA Segment A	0.511	[0.413,0.609]	<0.001
FONASA Segment B	0.282	[0.188,0.376]	<0.001
FONASA Segment C-D	0.206	[0.106,0.306]	<0.001
RM	-0.152	[-0.197,-0.107]	<0.001

The Cox model enables us to evaluate a patient’s survival rate, as identified through these eight selected covariates. These variables are private health insurance dummy variables, benefit segment A, B, and C-D dummy variables, age, squared age, year of diagnosis, and a dummy variable for residence in the Metropolitan area. The Cox model also allowed us to compare the survival curves by modifying selected variables while keeping the rest constant. For the results below, the unmodified variables are set as the median of numerical variables and the mode of categorical variables, which corresponds to a woman aged 57 years old who lives outside the Metropolitan region, was diagnosed in 2013, and is a beneficiary of segment B in the public healthcare system.

In the final model, all variables passed the proportional hazards assumption test; that is, no variable showed strong evidence of time dependence (p<0.001).

Hazard ratios (or odds ratios) between two sets of characteristics can be evaluated through Cox regression. Table 4 shows the hazard ratio for some specific variables. We observe that women 40 and 50 years old have almost the same hazard and that women 60 years old have a slightly higher hazard of dying due to breast cancer than women 40 years old. The hazard ratios for a difference of 10 and 20 years of age increase; thus, we observe that the hazard of 60–year–old women is 1.15 times that of 50–year–old women and that the hazard of 70–year–old women is 1.52 times higher that of 50–year–old women. The hazard of FONASA beneficiaries from the benefit segment C-D is 1.64 times that of ISAPRE beneficiaries. FONASA patients from segment B have a slightly higher hazard than FONASA patients from segments C-D. FONASA patients who benefit from segment A have an even higher hazard, 1.26 times higher than FONASA patients who benefit from segment B. Women diagnosed in 2018 have almost half the hazard (0.57) of women diagnosed in 2007.

**Table 4:** Hazard ratios obtained through the Cox model.

Coefficient	Hazard ratio
Age 40 → Age 50	0.99
Age 40 → Age 60	1.14
Age 50 → Age 60	1.15
Age 50 → Age 70	1.52
ISAPRE → FONASA Beneficiary C-D	1.64
FONASA Beneficiary C-D → FONASA Beneficiary B	1.08
FONASA Beneficiary B → FONASA Beneficiary A	1.26
Year 2007 → Year 2010	0.86
Year 2007 → Year 2013	0.74
Year 2007 → Year 2018	0.57
Region RM → Other Regions	1.16

**Fig 6:**
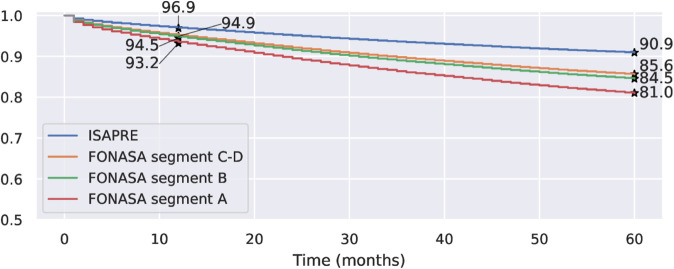
Cox survival curves adjusted by health insurance: ISAPRE (private) and FONASA (public) by segment. **Alt text:** The figure shows four stepped curves, showing the Cox estimated survival rates over time from 0 to 60 months. These four curves are the survival rates for patients from ISAPRE (private), C-D FONASA segment, B FONASA segment, and A FONASA segment. The four curves are descending, starting with a survival of 100% at time 0. The ISAPRE curve is above all FONASA segment’s curves at all times. The C-D Fonasa segment is very close to the B Fonasa segment at the beginning of the period, but has higher survival rates in the second half of the period. The A Fonasa segment sits below all other curves at all times. The estimated one-year and five-year survival rates are highlighted for each curve.

Fig 6 presents the Cox survival curves for women affiliated with public and private health insurance systems. We observe that the survival rate trends are similar to those obtained by the Kaplan–Meier estimations, with a survival curve for the patients in the private system significantly higher than that for patients affiliated with public health insurance. The survival curve for patients from segments C-D is slightly better than that of those from segment B. The curve for the FONASA patients from segment A is worse than the other curves. The Cox regression estimates the one-year survival rate to be0.969 for the private sector. The one-year survival rates for FONASA patients from segments A, B, and C-D are 0.932, 0.945, and 0.949, respectively. On the other hand, the Cox regression estimates the five-year survival rates to be 0.909 for the private system and 0.810, 0.845, and 0.856 for FONASA segments A, B, and C-D, respectively.

**Fig 7:**
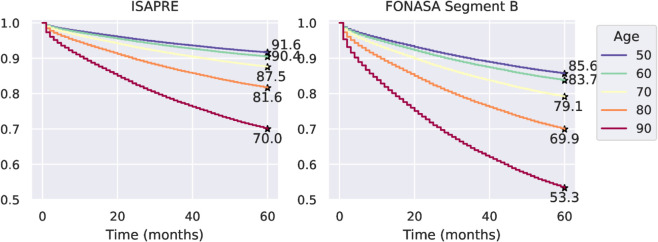
Survival curves obtained by Cox regression, adjusted by age, for each type of health insurance. **Alt text:** Figure shows two graphs. Each graph comprises five stepped curves, showing Cox estimated survival rates from 0 to 60 months. The left graph shows survival curves for ISAPRE patients, and the right graph shows survival curves for FONASA patients. The five curves in each graph show the Cox estimated survival curves for women aged 50, 60, 70, 80, and 90. All five curves in both graphs are descending, starting with a survival of 100% at time 0. The 90-year-old curve is below the 80-year-old curve at all times; the 80-year-old curve is below the 70-year-old curve at all times; the 70-year-old curve is below the 60-year-old curve at all times; the 60-year-old curve is below the 50-year-old curve at all times. The estimated five-year survival rates are highlighted in both graphs for each curve.

Fig 7 shows the survival curves for women of different ages in the private health insurance and public system, considering women from segment B. We noticed that the difference in survival rates between ISAPRE and B-segment FONASA patients increased for older people. The five-year survival rates for women aged 50 were 0.92 and 0.86 for the ISAPRE and FONASA systems, respectively, with a survival difference of 0.06. On the other hand, the five-year survival rates for women aged 80 are 0.82 for ISAPRE and 0.70 for FONASA segment B, with a survival difference of 0.12. Table 5 shows the five-year survival rates.

**Table 5:** Five–year survival rate predicted by the Cox model by age and health care system.

Age	50	60	70	80	90
ISAPRE	0.916	0.904	0.875	0.816	0.700
FONASA Segment B	0.856	0.837	0.791	0.699	0.533

## Discussion

In 2004, the Universal Access Plan for Explicit Guarantees (AUGE), later renamed GES, was introduced, a significant reform for the Chilean health system. This plan aimed for a quality healthcare system that could timely attend to all Chilean residents without discrimination and with financial protection. This provided access, quality, financial, and opportunity guarantees for a specific set of pathologies, which included breast cancer from its beginning in 2005. It defined the complete protocol of medical treatments, including procedures and drugs to diagnose and treat breast cancer in all its stages. It also defined medical protocols for the diagnosis and treatment of breast cancer in all its stages for women aged 15 years and over with suspected, diagnosed, or recurrent breast cancer [16]. Furthermore, the Ministry of Health currently *recommends* the realization of mammograms, aiming for early breast cancer detection. Nowadays, an annual mammogram is recommended for women 50 to 69 years old (www.diprece.minsal.cl). However, it only provides coverage for triannual screening mammograms for women aged 50 to 59 years old, independent of their health insurer [16].

There is evidence that women in the private system undergo more screening mammograms than those in the public system. This is illustrated by the National Socioeconomic Characterization Survey, CASEN 2017 [8], which shows that only 53.4% of women aged 50 or more in the private health system had a screening mammogram during the last year, while the percentage drops to 33.1% for women in the public system. These figures increase to 76.3% and 57.1% for a screening mammogram in the previous three years for the private and public sectors, respectively.

An essential step to change these results has been taken by MINSAL, which has made significant investments in equipment and human resources to improve breast cancer screening mammogram coverage. This investment has been primarily focused on local Family Health Centers (CESFAM) and community hospitals, along with the *Digital Hospital* program, where exams are taken by medical technologists in these healthcare facilities and are sent electronically to radiologists specializing in breast imaging. This system accounts for 22% of the mammography reports in the country from January to July 2021.

Even so, we remark that the Chilean Health system does not have a screening program as defined by the World Health Organization [17]. This is because there is a lack of an information system that can offer exams to eligible populations, utilize call and recall systems, and maintain accurate screening records.

Our findings underscore that survival disparities in breast cancer are not solely clinical but deeply rooted in structural and socioeconomic inequalities. They suggest that further investments in early detection infrastructure, particularly in public and low-complexity hospitals, could significantly improve outcomes. Expanding the GES coverage for mammography and strengthening referral pathways to high-complexity centers may reduce delays in diagnosis and treatment, particularly for underinsured populations.

In what follows, we present the main findings of our research. For this, we divide the discussion into three areas: estimates of case fatality ratios and survival rates, the impact of the GES plan on key healthcare indicators, and limitations and future research of this project.

### Case fatality ratios and survival rates

Our study estimates a national mean case fatality ratio of 26.8%, with a consistent trend throughout the study period. There is a high variation among regions, with no precise distribution. More importantly, there is a clear difference in the case fatality ratios of FONASA and ISAPRE, with averages for the 2007- 2018 period of 27.5% and 15.7%, respectively. This is because patients with public insurance have a fatality ratio that is 1.75 times higher than those with private insurance.

We estimated the five-year survival to be 0.829 (±0.004) at the national level. We estimated five-year survival rates of 0.808 (±0.004) and 0.902 (±0.007) for the public and private healthcare sectors, respectively.

Survival differences were also observed between patients from the Metropolitan region and those from other areas, both for the public and private healthcare systems. Patients from the Metropolitan region had better survival rates. This difference may be associated with the availability of specialized healthcare centers and specialists, which are known to be concentrated in metropolitan areas [18]. The five-year survival rates for patients in the Metropolitan region are 0.822 (±0.006) and 0.907 (±0.008) for women in FONSASA and ISAPRE, respectively. On the other hand, the five-year survival for patients from other areas are 0.789 (±0.006) and 0.892 (±0.012) for women in FONSASA and ISAPRE, respectively.

As expected, the hazard ratios obtained in our study indicate that age has a significant impact on survival. Thus, we observe that the hazard ratio for a 20–year difference between 40- and 60-year-old patients is 1.14 (that is, patients of 60 years old have a slightly higher risk of death due to breast cancer), while the same 20-year difference between 50- and 70-year-old patients leads to a hazard ratio of 1.52. It is also relevant to highlight the hazard ratio between private and publicly insured patients from segment C-D of 1.64, indicating that patients from the C-D segment in the public healthcare system have a risk 1.6 times higher than those from the private healthcare system.

Hazard ratios associated with the year of diagnosis show improved survival from 2007 onward. This may be attributable to advances in breast cancer treatment due to the system organization and the effect of the healthcare reforms implemented in previous years. Soon, we can probably observe that survival rates will continue to improve due to the implementation of the Ricarte Soto Law, which has created a financial protection system to cover high-cost diagnostic and treatment procedures, including those for Her-2 positive breast cancer [19].

By examining the hazard ratios between the Metropolitan region and other regions, we observe that women in the Metropolitan region have a slightly lower risk than women from other regions.

### GES plan and health care inequities

Most of the GES plan evaluations have focused on compliance with the explicit guarantees offered by the plan: access, opportunity, financial protection, and quality [11]. In terms of opportunity, in 2017, 99.6% of the GES services fulfilled this guarantee, with 92.8% of them meeting the established deadline. On the other hand, inspections by the Comptroller General of Chile (CGR) ensure that the healthcare facilities fulfill a series of quality measures. However, ultimately, the effectiveness of the GES must be reflected in improving health indicators, such as patient survival rates and case fatality ratios. As discussed in the Results section, we observed a marked difference in survival rates between insured patients in the public and private health systems. Thus, according to the Cox regression, at 50, the five-year survival rates for FONASA and ISAPRE patients were 0.86 and 0.92, respectively, with a difference of 0.06. Meanwhile, at 80, the five-year survival rates for FONASA and ISAPRE patients were 0.70 and 0.82, respectively, increasing the difference to 0.12. All these differences were statistically significant (p<0.001).

Multiple reasons contribute to this inequity between private and publicly insured patients. In what follows, we discuss some of them; however, they should be investigated in greater depth to develop and implement appropriate public policies to reduce this gap.

- ISAPRE and FONASA patients differ in their choice of health insurer and conform to two distinct sociodemographic group norms regarding socioeconomic status, education, and eventually comorbidities and age. For example, more than 85% of the people from the five lower income deciles belong to FONASA, while only 25% of people in the highest income decile belong to FONASA [8]. Likewise, income is also related to education, where the three lower income deciles have an average of less than 10 years of schooling, while people from the highest decile have an average of more than 15 years of schooling [20]. These differences may translate into variations in the opportunity to consult a doctor, treatment adherence, and treatment outcomes, particularly due to comorbidities.
- The GES plan guarantees an adequate diagnosis and treatment, as defined in clinical guidelines, and is periodically updated to incorporate new validated therapeutic options. However, there might be a lag in the opportunity of when these updates are incorporated. For example, the high-cost drug trastuzumab was included for all Chilean women with HER2-positive breast cancer in 2015, nine years after it was approved for use by the FDA. Consequently, although quality treatment is guaranteed for all breast cancer patients, there is a time gap between the appearance of new drugs adequately supported by sound evidence and their incorporation in the GES plan. Therefore, patients with higher purchasing power are most likely to complement their treatments with these new drugs outside of the GES plan, leading to higher survival rates. In addition, ISAPRE patients who belong to the highest income groups often have extra complementary insurance provided by insurance companies that they pay out of pocket, which further increases the coverage of high-cost drugs.
- Early detection is a key factor for survival rates: cancer detected in stages 1 and 2 has a five-year survival rate of 99%. In contrast, survival rates when cancer is detected in stages 3 and 4 are 86% and 28%, respectively [21]. According to data from 2017, 55.1% of women in the public health system had a screening mammogram in the last three years compared to 71.4% for women in the private system [8], which might translate into significant inequities in the cancer stage at the moment of diagnosis between women in the two insurance systems. As mentioned before, Chile does not have a national cancer registry, and therefore, staging of breast cancer at diagnosis is not available to confirm this hypothesis. As part of our ongoing research, we are estimating cancer staging at diagnosis using databases of private and public hospitals.

### Study strength and limitations

To the best of our knowledge, this is the first study on breast cancer fatality and survival rates at the national level using publicly available data from several national registries on death records and hospital discharges. Additionally, this study presents key healthcare indicators for women across various regions, healthcare insurance plans, and age groups.

This study has several limitations. First, the quality and completeness of the national hospital discharge and mortality databases may affect the accuracy of our findings. As previously noted and discussed in [1], these limitations could be mitigated by establishing a national cancer registry in Chile. Additionally, our analysis relies on hospital discharge data, which includes all discharges from public and private healthcare facilities nationwide. However, some breast cancer patients—particularly those not undergoing surgery—may receive outpatient care only and thus not be captured in this dataset. Consequently, case fatality ratios may be overestimated. Moreover, the exclusion of non-hospitalized patients introduces potential bias in survival estimates, as these individuals may differ systematically in clinical stage, comorbidities, or socioeconomic status.

Regarding external validity, while the study offers valuable insights into the Chilean context, generalizing the findings beyond this setting requires caution. The sample includes women treated in public and private hospitals in Chile, which may not reflect the full spectrum of healthcare experiences across all regions or health facilities. Furthermore, healthcare systems in other countries may differ substantially in structure, financing, and access barriers, limiting the applicability of our conclusions internationally.

As part of our future research, we aim to track breast cancer outcomes in relation to recent healthcare updates and investments, particularly those focused on improving mammography access and care coordination. For example, as part of the Digital Hospital strategy led by Chile’s Ministry of Health, a telemammography project was launched in 2021 to improve early breast cancer diagnosis in areas with limited access to specialists. By enabling the remote transmission of mammograms through 5G networks, the initiative significantly reduced image transfer and interpretation times. To date, the program has processed over 100,000 mammograms, enabling primary care centers to digitally refer patients for specialist consultations at the Digital Hospital. This initiative has helped decentralize care, expand diagnostic coverage, and reduce disparities in early breast cancer detection within the public healthcare system. As part of our research, we are working to quantify whether or not this initiative has had an impact on reducing the disparity observed in the present study.

## Conclusions

The methodology employed in this study provides an alternative approach for obtaining case fatality ratios and survival rates from public databases, which are key measures of importance, particularly in the absence of a national cancer registry, as is the case in Chile.

The reasons that explain the significant differences in both rates, especially between private and publicly insured patients, are part of our ongoing research. However, these results have already highlighted significant inequalities in health outcomes between these two patient groups. This is particularly relevant when a new health system is being discussed in Chile.

## Supporting information

S1 Table Fonasa survival records. The Kaplan–Meier curves for women in FONASA are made with the observed and censored events shown in this table.
(PDF)

S2 Table Isapre survival records. The Kaplan–Meier curves for women in ISAPRE are made with the observed and censored events shown in this table.
(PDF)

S3 Table Chilean survival records. The Kaplan–Meier curves for all women in Chile are made with the observed and censored events shown in this table.
(PDF)

S1 Appendix Cox regression analysis. (PDF)
