# Supplementary material for: Inequities in breast cancer outcomes in Chile: An analysis of case fatality ratios and survival rates (2007–2018)
Source: PLoS One. 2025 Sep 29;20(9):e0325252. doi: 10.1371/journal.pone.0325252 (PMC12478957; doi:10.1371/journal.pone.0325252)
Supplement: S1 Table — The Kaplan–Meier curves for women in FONASA are made with the observed and censored events shown in this table. (PDF) [file pone.0325252.s001.pdf]

S1 Table: Fonasa Survival Records

The Kaplan–Meier curves for women in FONASA are made with the observed and censored events shown in this table. The event table for ISAPRE and all women in Chile can be found in S2 and S3 Tables, respectively. Event tables for other Kaplan–Meier curves shown in the results section are not included due to their extension.

Table 1. Event table for the survival curve for patients in the public health system, months 0 through 30.

Time (months)	Removed	Observed	Censored	At risk
0	0	0	0	43025
1	796	712	84	43025
2	692	333	359	42229
3	614	247	367	41537
4	545	195	350	40923
5	466	189	277	40378
6	508	142	366	39912
7	516	187	329	39404
8	537	157	380	38888
9	499	142	357	38351
10	483	134	349	37852
11	550	154	396	37369
12	456	153	303	36819
13	475	140	335	36363
14	437	128	309	35888
15	440	129	311	35451
16	433	121	312	35011
17	427	126	301	34578
18	469	114	355	34151
19	434	121	313	33682
20	479	134	345	33248
21	482	128	354	32769
22	389	114	275	32287
23	477	112	365	31898
24	444	137	307	31421
25	427	104	323	30977
26	444	87	357	30550
27	418	109	309	30106
28	423	105	318	29688
29	376	88	288	29265
30	418	93	325	28889

Table 2. Event table for the survival curve for patients in the public health system, months 31 through 60.

Time (months)	Removed	Observed	Censored	At risk
31	425	104	321	28471
32	349	91	258	28046
33	415	89	326	27697
34	408	73	335	27282
35	400	91	309	26874
36	376	89	287	26474
37	393	63	330	26098
38	333	56	277	25705
39	410	74	336	25372
40	392	63	329	24962
41	352	72	280	24570
42	402	69	333	24218
43	378	59	319	23816
44	385	59	326	23438
45	339	66	273	23053
46	355	69	286	22714
47	379	59	320	22359
48	342	65	277	21980
49	356	49	307	21638
50	295	52	243	21282
51	322	61	261	20987
52	340	52	288	20665
53	314	54	260	20325
54	333	42	291	20011
55	282	48	234	19678
56	288	45	243	19396
57	327	46	281	19108
58	290	36	254	18781
59	312	51	261	18491
60	18179	42	18137	18179
