# Supplementary material for: Inequities in breast cancer outcomes in Chile: An analysis of case fatality ratios and survival rates (2007–2018)
Source: PLoS One. 2025 Sep 29;20(9):e0325252. doi: 10.1371/journal.pone.0325252 (PMC12478957; doi:10.1371/journal.pone.0325252)
Supplement: S2 Table — The Kaplan–Meier curves for women in ISAPRE are made with the observed and censored events shown in this table. (PDF) [file pone.0325252.s002.pdf]

S2 Table: Isapre Survival Records

The Kaplan–Meier curves for women in ISAPRE are made with the observed and censored events shown in this table. The event table for FONASA and all women in Chile can be found in S1 and S3 Tables, respectively. Event tables for other Kaplan–Meier curves shown in the results section are not included due to their extension.

Table 1. Event table for the survival curve for patients in the private health system, months 0 through 30.

Time (months)	Removed	Observed	Censored	At risk
0	0	0	0	11147
1	70	64	6	11147
2	143	33	110	11077
3	140	14	126	10934
4	143	31	112	10794
5	109	29	80	10651
6	141	20	121	10542
7	105	23	82	10401
8	94	13	81	10296
9	107	17	90	10202
10	110	15	95	10095
11	101	17	84	9985
12	83	12	71	9884
13	104	11	93	9801
14	119	15	104	9697
15	112	17	95	9578
16	119	9	110	9466
17	132	20	112	9347
18	112	15	97	9215
19	116	15	101	9103
20	129	19	110	8987
21	99	15	84	8858
22	78	11	67	8759
23	96	11	85	8681
24	68	13	55	8585
25	117	23	94	8517
26	121	9	112	8400
27	126	16	110	8279
28	113	16	97	8153
29	116	11	105	8040
30	133	12	121	7924

Table 2. Event table for the survival curve for patients in the private health system, months 31 through 60.

Time (months)	Removed	Observed	Censored	At risk
31	110	4	106	7791
32	112	10	102	7681
33	119	18	101	7569
34	115	10	105	7450
35	127	9	118	7335
36	76	10	66	7208
37	126	7	119	7132
38	109	18	91	7006
39	120	13	107	6897
40	88	11	77	6777
41	85	10	75	6689
42	95	14	81	6604
43	96	6	90	6509
44	102	14	88	6413
45	105	19	86	6311
46	90	10	80	6206
47	119	8	111	6116
48	76	8	68	5997
49	109	8	101	5921
50	105	7	98	5812
51	101	8	93	5707
52	122	12	110	5606
53	67	6	61	5484
54	86	8	78	5417
55	81	8	73	5331
56	97	6	91	5250
57	73	6	67	5153
58	94	6	88	5080
59	91	11	80	4986
60	4895	11	4884	4895
