# Supplementary material for: Inequities in breast cancer outcomes in Chile: An analysis of case fatality ratios and survival rates (2007–2018)
Source: PLoS One. 2025 Sep 29;20(9):e0325252. doi: 10.1371/journal.pone.0325252 (PMC12478957; doi:10.1371/journal.pone.0325252)
Supplement: S3 Table — The Kaplan–Meier curves for all women in Chile are made with the observed and censored events shown in this table. (PDF) [file pone.0325252.s003.pdf]

S3 Table: Chilean Survival Records

The Kaplan–Meier curves for all women in Chile are made with the observed and censored events shown in this table. The event table for FONASA and ISAPRE can be found in S1 and S2 Tables, respectively. Event tables for other Kaplan–Meier curves shown in the results section are not included due to their extension.

Table 1. Event table for the survival curve for all patients in the chilean health system, months 0 through 30.

Time (months)	Removed	Observed	Censored	At risk
0	0	0	0	58235
1	908	815	93	58235
2	882	384	498	57327
3	810	283	527	56445
4	739	243	496	55635
5	619	229	390	54896
6	702	170	532	54277
7	680	226	454	53575
8	679	184	495	52895
9	655	169	486	52216
10	638	161	477	51561
11	689	175	514	50923
12	573	170	403	50234
13	620	162	458	49661
14	596	150	446	49041
15	587	159	428	48445
16	589	134	455	47858
17	597	159	438	47269
18	621	140	481	46672
19	585	141	444	46051
20	651	162	489	45466
21	610	148	462	44815
22	497	135	362	44205
23	613	131	482	43708
24	559	158	401	43095
25	600	139	461	42536
26	604	102	502	41936
27	590	136	454	41332
28	568	127	441	40742
29	531	104	427	40174
30	588	111	477	39643

Table 2. Event table for the survival curve for all patients in the chilean health system, months 31 through 60.

Time (months)	Removed	Observed	Censored	At risk
31	559	112	447	39055
32	485	110	375	38496
33	569	111	458	38011
34	548	88	460	37442
35	567	112	455	36894
36	481	105	376	36327
37	552	80	472	35846
38	486	80	406	35294
39	564	97	467	34808
40	504	76	428	34244
41	467	89	378	33740
42	539	90	449	33273
43	497	70	427	32734
44	526	85	441	32237
45	482	91	391	31711
46	482	83	399	31229
47	526	74	452	30747
48	432	76	356	30221
49	491	65	426	29789
50	431	65	366	29298
51	449	72	377	28867
52	494	68	426	28418
53	395	64	331	27924
54	445	53	392	27529
55	396	58	338	27084
56	406	53	353	26688
57	419	55	364	26282
58	408	47	361	25863
59	438	62	376	25455
60	25017	55	24962	25017
