# Supplementary material for: Inequities in breast cancer outcomes in Chile: An analysis of case fatality ratios and survival rates (2007–2018)
Source: PLoS One. 2025 Sep 29;20(9):e0325252. doi: 10.1371/journal.pone.0325252 (PMC12478957; doi:10.1371/journal.pone.0325252)
Supplement: S1 Appendix — (PDF) [file pone.0325252.s004.pdf]

S1 Appendix: Cox Regression Analysis

The variables were processed before selection. We added squared age as a new variable to add more flexibility to hazard modeling as a function of age and normalized both age and squared age to a maximum age of 100 years. The categorical health insurance and FONASA benefit segment variables were transformed into dummy variables. Following results from the previous section, a dummy variable that considered both C and D FONASA benefit segments was included to add freedom to the model. For simplicity, the region of residence variable was reduced to a dummy variable indicating if the patient had a residence in the Metropolitan region. This process resulted in a total of 13 covariables: 4 health insurance dummy variables accounting for public, private, armed forces, and missing healthcare insurance; 5 benefit segment dummy variables, namely segments A, B, C, D, and C-D segments; 1 region dummy variables indicating residence in the Metropolitan area; year of diagnosis; normalized age and squared normalized age.

The selection of variables was performed using a greedy algorithm based on Akaike's information criterion and the p-value for the significance of each variable. That is, we sequentially selected the variables by adding those that mainly improved Akaike's information criterion while maintaining the p-values of all variables as statistically significant. The selection also considered the exclusion of non-independent variables, this is, if we included the variables for C or D FONASA segments, we could no longer add the combined C-D FONASA segment, for the last would no longer be independent of the previous variables.

More formally, given a set of n independent variables from which to choose, we proceeded based on each variable's p-value and the model's Akaike information criterion as follows.

Pseudocode for greedy variable selection for Cox model.

Variable selection:
Set the current best variables as an empty list.
Set the current best Akaike value as infinity.
Set the n variables as a pending list.
While there are still variables in the pending list, do the following:
For each variable from the pending list, generate a Cox model with it and the selected variables.
For each such model, check if all their variables have significant p values (≤0.001). If none of the models comply with it, break.
For each model that meets the above:
Calculate the Akaike information criterion.
If the calculated Akaike value is lower than the current best, set such value as the current best Akaike value and add such variable to the current best variables, removing it from the pending list.
Break if none of the above models achieves an Akaike value better than the current best.
Return the model generated with the current best variables.

For the 13 variables mentioned above, the previous process selected the following 8 variables: normalized age, square normalized age, private health insurance dummy, discharge year, A FONASA beneficiary segment dummy, B FONASA beneficiary segment dummy, C-D FONASA beneficiary segment dummy, residence in the Metropolitan region dummy.
